# A Team Approach: How the LEND Program Can Provide Interdisciplinary Training for Behavior Analysts

**DOI:** 10.1007/s40617-025-01043-2

**Published:** 2025-02-07

**Authors:** Lori B. Vincent, Meagan Scott, Stephanie Weber, Laura Srivorakiat, Mary Stepanek, Jennifer Smith

**Affiliations:** 1 School of Human Services, School Psychology Programs, University of Cincinnati, 450B Teacher-Dyer Complex, Cincinnati, OH 45221 USA; 2 Department of Educational, School, and Counseling Psychology, University of Kentucky, Lexington, KY USA; 3 Department of Pediatrics, College of Medicine, University of Cincinnati, Cincinnati, OH USA; 4 Cincinnati Children’s Hospital Medical Center, Cincinnati, OH USA

**Keywords:** LEND program, Applied behavior analysis, Neurodevelopmental disabilities, Autism, Interdisciplinary work, Training

## Abstract

Supporting people with neurodevelopmental disabilities often requires interdisciplinary collaboration and effective partnerships with clients and their families. Behavior analysts receive intensive training and supervision in a variety of domains; however, expanding interdisciplinary training for behavior analysts is needed. Interdisciplinary training programs, such as the Leadership Education in Neurodevelopmental and Related Disabilities (LEND) programs, offer trainees the opportunity to hone their skills as advocates for people with neurodevelopmental disabilities and collaborators as part of an interdisciplinary team. Historically, many LEND programs have not offered training positions specifically to behavior analysis students or professionals, although some behavior analysts have participated as trainees in other disciplines such as psychology or special education. The benefits, barriers, and possible future directions of interdisciplinary training for behavior analysts within a LEND program are discussed through the experience of the University of Cincinnati LEND Program which added an Applied Behavior Analysis (ABA) training track in 2020.

Interdisciplinary treatment involves professionals from different disciplines who each maintain their respective disciplinary role, work together to coordinate tasks and care for a client, have frequent communication and collaboration about the treatment plan and progress, and have shared goals and responsibilities (Bowman et al., 2021). Interdisciplinary treatment is often considered valuable for individuals with complex needs, including people with neurodevelopmental disabilities (NDD), although more research in this area is needed (Bowman et al., 2021; LaFrance et al., 2019).

Key characteristics of interdisciplinary teams include a shared philosophy, interdependence of team members, professional identity based on relevant knowledge rather than title, leadership skills within the team, dynamic and evolving collaboration, and a partnership, based on respect and trust, that uses strong communication skills to combine the knowledge of others (Kunze & Machalicek, 2022). Although professionals in the field of applied behavior analysis (ABA) provide valuable services to people with NDD, they may at times face cross-disciplinary challenges (e.g., use of different terminology, misconceptions and misunderstandings between disciplines) that may affect their collaboration with other providers compared to other professionals in related fields (Brown & Hendy, 2001; Slim & Reuter-Yuill, 2021). This may be due to the isolation of behavior analysts in their training compared to other disciplines (Brown & Hendy, 2001; Kelly & Tincani, 2013) and the training some behavior analysts receive related to practices considered not evidence-based used in other disciplines such as occupational therapy (Leaf et al., 2023). Therefore, effective pre-service training and increased opportunities for interdisciplinary work is essential to enhance the skillsets of ABA providers (Kirby et al., 2022) and improve the overall interdisciplinary care of individuals with NDD and their families (Hall, 2005). When implemented effectively, interdisciplinary care can improve the family-centered coordinated care that children receive (Rohrer et al., 2021; Suen et al., 2021); however, there are still a multitude of challenges and barriers that hinder the effectiveness of interdisciplinary care including cost of services and billing (Bowman et al., 2021), which are beyond the scope of this paper.

## Challenges and Barriers to Interdisciplinary Work and Training

Research has identified many challenges and barriers that may impact success in meaningful and productive interdisciplinary collaboration. First, practitioners from different disciplines may vary in their core values and definitions of evidence-based practices (EBP), which can lead to contrasting opinions and recommendations (Bowman et al., 2021; Gasiewski et al., 2021). Additionally, misunderstanding others’ roles can often lead to less collaboration and more conflict (Gasiewski et al., 2021). For example, negative views or misperceptions of other disciplines can result in a lack of trust and respect within an interdisciplinary team (Bowman et. al, 2021; Gasiewski et al., 2021). Finally, interdisciplinary teams face challenges when providers engage in disciplinary-centric attitudes or belief that one’s discipline is superior and better equipped to address client concerns (Pecukonis, 2020).

Specifically, ABA providers may find it difficult to collaborate with other disciplines and eventually limit collaborations due to a misalignment between the fundamental dimensions of ABA and other disciplines’ practices (Baer et al., 1968; Cooper et al., 2019; Welch & Polatajko, 2016). For instance, ABA providers may have negative preconceived notions about other providers use of nonbehavioral or non-evidence-based interventions that are perceived to lack systematic instruction, data collection, or individualization (Gasliewski et al., 2021; Lane & Brown, 2023). This may stem from ABA providers being less likely to read the literature from other disciplines related to NDD than their colleagues from different disciplines (Smith, 2012), thus resulting in ABA providers relying on their own training, opinions, and biases. Similarly, other disciplines may not agree with behavioral-based interventions due to them being perceived as rigid or ineffective, disregarding clients’ needs, and undervaluing diversity in individual functioning (Lane & Brown, 2023; Welch & Polatajko, 2016). Additionally, differences in terminology and jargon across disciplines can lead to ineffective communication (Bowman et al., 2021). This may be especially true for behavior analysts as the field of ABA has created unambiguous terminology that unifies the work of behavior analysts yet isolates behavior analysts from other disciplines (Brown & Hendy, 2001). Interdisciplinary team barriers derive from multiple perspectives, and these barriers can and should be addressed to increase effective cross-disciplinary collaboration and quality care.

The Behavior Analyst Certification Board’s (BACB, 2020) Ethical Codes 2.10 requires ABA providers to collaborate with other providers for the best interest of the client as appropriate. Although the need for interpersonal and technical skills training for ABA providers is imperative to the field of ABA (Taylor, et al., 2019), ABA providers often receive a limited amount of training in collaboration and interdisciplinary work (Kelly & Tincani, 2013). For instance, research has shown that ABA providers lack training in “soft skills,” such as perspective taking and compassionate responding (Taylor et al., 2019). Additionally, a personality survey found that ABA students demonstrated lower levels of warmth (i.e., warm, outgoing, attentive to others) than students within other human service disciplines (Callahan et al., 2019). This is especially important as behavior analysts who demonstrated high levels of caring interpersonal skills were more highly rated by parents of children receiving ABA services (Callahan et al., 2019).

Without specific education and/or training in collaborative practice, interdisciplinary collaboration may present challenges to behavioral health providers. Interdisciplinary collaboration is rarely observed in academia and interactions among students of differing professions are not necessarily encouraged or facilitated (Kelly & Tincani, 2013). This lack of interpersonal collaboration training can lead to distinct and narrow professional worldviews where providers embrace their professional identity and continue to create silos of care due to only collaborating with providers within the same field (Pecuknois, 2020; Weiss et al., 2022). Additionally, effective collaborate between ABA providers and other disciplines may assist in avoiding unnecessary undesirable outcomes, including poor social validity of interventions and negative and/or traumatic experiences of clients when that integrated care is provided ethically (Cox, 2012, 2019; Kupferstein, 2018; Sandoval-Norton & Shkedy, 2019).

## Benefits of Interdisciplinary Teams

To address the lack of training in interdisciplinary work and barriers to interdisciplinary collaboration, ABA providers would likely benefit from increased opportunities to work on interdisciplinary teams. Collaborating within an interdisciplinary framework can help ABA providers recognize, understand, value, and appreciate the contributions of professionals from other disciplines (LaFrance et al., 2019). For example, interdisciplinary training allows ABA trainees to improve their interpersonal skills in working with families, clients, and other professionals by creating opportunities for ABA trainees to observe the interpersonal skills of professionals from other disciplines and learn from their interactions with families and clients.

Experience working on interdisciplinary teams can also help practitioners hone their communication skills to better communicate with providers from other disciplines. Specifically, ABA providers can learn to use clear communication to share knowledge, exchange ideas, and effectively coordinate care for clients (Bowman et al., 2021). When ABA providers develop effective communication skills, they may be more likely to resolve conflicts and misunderstandings that occur within interdisciplinary teams with various clinical perspectives quickly, effectively, and respectfully. In addition, having direct experiences can help practitioners identify their role within a team setting and create clear professional boundaries between all interdisciplinary team members (Bowman et al., 2021).

## Interdisciplinary Training Components

Interprofessional training for ABA providers could include opportunities to practice sharing basic information about each discipline, observe other disciplinary providers, provide cotreatment sessions, participate in interdisciplinary case conferences, and read research published by different disciplines (Gasiewski et al., 2021). Additionally, during the interdisciplinary training, ABA providers must learn how to effectively query and consult with other providers, especially when there are discussions about the implementation of other disciplines’ evidence-based interventions (Gasiewski et al., 2021). It is essential for ABA providers to learn to value the contributions and input from other disciplines, as this may.

ultimately encourage them to acknowledge and address professional mistakes and/or areas for growth (Wright, 2019). This might help ABA providers improve their relationships with clients, families, and other professionals. If ABA providers grow their humility in their professional practice, it may also allow them to be more compassionate and understanding of other providers on the team when mistakes are made. This compassion and understanding fosters trust and mutual respect (Gasiewski et al., 2021). For the overall wellbeing of the client, it is important that interdisciplinary teams learn to focus on the shared goals and commonalities, rather than on individual differences between disciplines.

There are several promising models of collaborative training for ABA providers. One strong example is the Melmark’s Collaboration Learning Series. In this training, ABA providers are exposed to literature from all disciplines, receive mentorship on case studies from providers in other disciplines, receive feedback on their interactions with providers from other disciplines, and conduct presentations on allied disciplines (Boivin et al., 2021). Including these activities in preservice training provides trainees with a firm understanding of the strengths and benefits of other disciplines, and the value of professional respect and interdisciplinary collaboration (Boivin et al., 2021).

Additionally, Kunze and Machalicek (2022) have proposed Professional Development Toward Interdisciplinary Teaming. This model includes three stages: (1) Initial Uni-Disciplinary Training, (2) Evolving Professional, and (3) Interdisciplinary Team Member. Uni-disciplinary training is the earliest stage of training which often includes university preparation to develop practical knowledge in a single discipline, allowing the trainee to develop an individual professional identity. The Evolving Professional stage occurs during early practice with supervision where the professional implements their new skills, identifies where they need additional training, and seeks further training and supervision as needed. In the Interdisciplinary Team Member stage, the professional embraces their area of expertise and recognizes the constraints of their training and practice in comparison with other disciplines. At this stage, the professional seeks interdisciplinary partners to enhance client outcomes and to further their own training from other disciplines (Kunze & Machalicek, 2022).

Bowman and colleagues (2021) have also proposed standards for collaborative practice to promote appreciation of each discipline’s expertise and to facilitate effective problem solving. They proposed eight specific standards: (1) collaborative communication; (2) distinguished roles in collaboration; (3) role of organization; (4) client care; (5) conflict resolution; (6) joint partnerships; (7) evidence-based practice; and (8) collaborative culture. While these frameworks provide strong guidance in interdisciplinary training for ABA providers, they do require significant effort to build and sustain these infrastructures within traditional training programs that may not have access to providers from other disciplines. Embedding training opportunities within programs that already provide quality interdisciplinary training can decrease the demand on single training programs. One such training opportunity is through the Leadership Education in Neurodevelopmental and Related Disabilities (LEND) programs.

## Maternal and Child Health (MCH) LEND Program Background

LEND programs, funded by the Maternal and Child Health Bureau (MCHB), include interdisciplinary training for individuals from a variety of disciplines. Trainees are often graduate students, early career professionals, family members, or self-advocates who participate in year-long immersive training to develop leadership skills in supporting people with NDD and their families. There are currently 60 LEND programs across the United States (Association of University Centers on Disabilities, 2022). Typical disciplines include audiology, genetic counseling, health administration, medicine, nursing, nutrition, occupational and physical therapy, pediatric dentistry, psychology, public health, social work, special education, and speech-language pathology (Keisling et al., 2017). Additionally, programs include family trainees (i.e., a family member of individual with an NDD), and self-advocate trainees (i.e., an individual with an NDD) with lived experience. The inclusion of people with lived experience like family members and self-advocates has been found to help clinical trainees from various professional disciplines increase their perceived knowledge, skills, and leadership development in family-centered care (Keisling et al., 2017). Previous research has found that future professionals who participated in a LEND program were more likely to find employment where they can support people with NDD and demonstrated stronger interdisciplinary practices (Rosenburg et al., 2015).

Although LEND programs have been providing interdisciplinary training since the late 1960s, only a few programs include ABA as a specific training discipline which has been a missed opportunity both for the field of ABA and LEND programs. On the basis of our review of the LEND website (Health Resources & Services Administration, 2024), of the 29 programs in which we could find a list of training disciplines on their funded project page or in the linked website, five listed Applied Behavior Analysis as a discipline. On the basis of our personal experience and knowledge of faculty and trainees past and present in other LEND programs, behavior analysis as a field has been represented in LEND programs, though often under another discipline such as psychology, special education, speech and language pathology, or occupational therapy. Following changes in insurance coverage of ABA services, state licensure/certification of behavior analysts, and increase higher education degree programs specifically in ABA, there have been a significant increase in jobs for behavior analysts (BACB, 2024; Yingling et al., 2022). Although behavior analysts may participate in many LEND programs, not having a specific discipline designation may limit the participation of behavior analysts who do not have training in an additional discipline or in the number of trainees from behavior analysis who can participate as trainees.

## Program Description

The Cincinnati LEND Program was one of the first LEND programs to be developed, launching in the late 1960s at Cincinnati Children’s Hospital. Prior to the 2020–2021 cohort, the Cincinnati LEND Program included the following disciplines: Audiology, Biomedical Engineering, Child Life, Community Engagement, Developmental-Behavioral Pediatrics, Disability Studies, Diversity, Family, Genetic Counseling, Nursing, Occupational Therapy, Organizational Leadership, Physical Therapy, Psychology, Self-Advocacy, Sibling, Social Work, and Speech-Language Pathology. Leadership training and interdisciplinary teamwork are both interwoven throughout all elements of Cincinnati LEND’s cohort model of professional development. All trainees participate in eight hours of synchronous, in-person classroom experiences weekly from August through April of the academic year. A minimum of 29 trainees are recruited to participate per year, representing at least 12 distinct disciplines. Cincinnati’s LEND curriculum consists of five training components: (1) Interdisciplinary Leadership Seminar (LEAD), (2) Interdisciplinary Training Team (ITT), (3) Core Course, (4) Seminar in Evidence-Based Methods (SEBM), and (5) Disciplinary Clinical Experiences. Each course and all projects are based upon one or more of the MCH Leadership Competencies, the 12 domains which define the necessary knowledge and skills for effective leadership in the NDD field (see Table 1; Maternal & Child Health Bureau, 2023). All team-based projects are established to have trainees paired or grouped with other disciplines. Additionally, at the beginning of the year, trainees within each discipline prepare and deliver an oral presentation to the larger group on their specific discipline, which includes the scope of practice in the field of developmental disabilities and training/licensure requirements.

**Table 1:** Select LEND training activities

LEND Course	Topic	Teaching method	Time
Interdisciplinary training team	ITT cases	Case-based learning, disciplinary presentations	20–25 h per team
Interdisciplinary training team	Observations across disciplines	Clinical observations, written reflections	8–12 h
Interdisciplinary training team	Supervision and management	Didactic, group discussion	2 h

Table from Weber et al., 2021

In the sections below, the LEND core competencies, development of the LEND ABA track, specific LEND training activities, alignment of LEND training experience with the BACB competencies, and recommendations to expand representation of behavior analysts in LEND programs will be discussed.

### LEND Core Competencies

The LEND program focuses on development and demonstration of core competencies, including (1) interdisciplinary work; (2); advocacy; (3) intersectional approach; (4) systems perspective; (5) life course perspective; (6) leadership; (7) engagement with maternal and child health populations; and (8) research experience (Bishop et al., 2023). As this paper focuses on interdisciplinary teamwork, in this section, the *Interprofessional Collaborative Practice Core Competencies* will be discussed. There are four core competency domains for interprofessional collaborative practice which emphasize knowledge, skills, attitudes, and values within collaborative practice (IPEC Expert Panel, 2011, p. 12; IPEC, 2016). The four *Interprofessional Collaborative Practice Competencies* of (1) values and ethics; (2) roles and responsibilities; (3) interpersonal communication; and (4) team and teamwork are described in Table 2. These competencies require professionals from different disciplines to respectfully engage in evidence-based practice and collaboration that incorporates shared values, goals, and decision-making (Bruder et al., 2019; LaFrance et al., 2019; Lane & Brown, 2023).

**Table 2:** Interprofessional collaborative practice core competencies

Core competency	Description
Value and ethics	Working with professionals from other disciplines to maintain a climate of mutual respect and shared values and to construct a culture that embodies a shared understanding of interprofessional education and collaborative principles. This also includes demonstrating cultural sensitivity and responsiveness, cultural humility, and cultural reciprocity
Roles and responsibilities	Acknowledgement of team members’ roles and abilities and development of a clear scope of practice and competence with respect to discipline specific competencies. Clear communication of roles is needed, along with self-reflection, ongoing clarity of roles and responsibilities of each team member, and development of an interdependent relationship
Interpersonal communication	Clear, understandable, and respectful communication across disciplines. Communication should express empathy and compassion and focus on resolution of conflicts
Teams and teamwork	Applying relationship and team-building values and principles while seeing each disciplines’ contribution as equally significant and meaningful (Bricker et al., 2022)

### Development of LEND ABA Training Track

On the basis of the feedback from previous Cincinnati LEND trainees, there was a desire for additional educational opportunities to learn about ABA. To address this need, a Board Certified Behavior Analyst – Doctoral Level (BCBA-D) psychologist was appointed as LEND faculty to provide additional ABA-related curriculum, hence leading to the creation of the ABA training track. The ABA-specific training track was piloted in the LEND program in 2020 when a trainee applied for a different discipline track but was also currently in training to become a behavior analyst. Each year, the training experience has been refined, including the addition of clinical training, supervision, and identifying the role of the ABA trainee in LEND specific activities which are each described below.

### Interdisciplinary Training Team (ITT)

This two-semester, team-based experience (60 h) uses the structure of a traditional interdisciplinary team approach to comprehensive assessment. The course begins with didactics on the comprehensive evaluation process for autism and other NDD and the basics of standardized testing including psychometric properties and common modifications that may be necessary in testing. Trainees discuss assigned readings regarding clinical assessment of young children and interdisciplinary team evaluations and the application of Life Course Perspective and Social Determinants of Health to the evaluation process. Next, trainees complete supervised disciplinary evaluations of children who present with possible autism or other NDD and/or observe evaluations by other disciplines which occur during morning LEND hours. Table 3 provides a list of training disciplines and the role of each discipline during the ITT assessment process. In afternoon ITT team meetings that occurred every other week, trainees discuss findings with all LEND disciplines represented and work together to develop a family-centered approach to the remaining evaluation visits, information sharing session, and follow-up with the family. This course results in frequent robust discussions among trainees and faculty regarding diagnoses, treatment options, and family priorities.

**Table 3:** Interdisciplinary training team

Discipline	Role and responsibilities
Applied Behavior Analysis	Functional Behavior Assessment (FBA) Caregiver Interview; preference assessment
Audiology	Hearing; speech discrimination; auditory responsiveness
Developmental-Behavioral Pediatrics	Medical history; family history; presenting developmental concerns; physical exam
Family	Family history; family strengths/challenges; family goals
Genetic Counseling	Family history risk assessment of genetic conditions; genogram
Occupational Therapy	Fine motor skills; sensory processing; activities of daily living
Physical Therapy	Gross motor skills; gait; coordination; balance
Psychology	Cognitive functioning; adaptive functioning; behavioral functioning; executive functioning; social-emotional functioning; autism specific measures and observations
Social Work	Psychosocial history; stressors; resources
Special Education	Academic functioning; review of Individualized Education Plan (IEP)
Speech Language Pathology	Expressive and receptive language; social-pragmatic skills; articulation

During the team meetings, all trainees can query and conceptualize the various disciplinary evaluation methodology and results. These meetings often result in conversations about clarifying the client’s concerns through the lens of all the participating clinical trainees and how to apply a family-centered approach during future follow-up with the client and their family. The ABA trainee participates in the ITT experience with two distinct clients and families during the LEND training year. Trainees rotate roles in the ITT process and their leadership skills in this process are evaluated by the LEND supervisors and staff.

Initially, the ABA trainee served in a nonclinical role where they participated in interdisciplinary observation and ITT team discussion but did not conduct an actual assessment. When the track started, ABA was not a discipline that participated in evaluations at the medical center which was the rationale for the initial clinical observer role. The ultimate goal of interweaving ABA into the ITT experience was to add the ABA trainee as a clinical, patient-facing discipline. On the basis of ABA trainee feedback over the initial training years, an ABA evaluation has been added solely for the purpose of the ITT. Depending on the client’s history and concern, the ABA trainee can conduct an indirect functional behavior assessment interview and/or survey with a caregiver, direct observation of the child’s behavior, and a preference assessment with the child. The ABA trainee prepares and delivers a presentation to their ITT which includes a discussion on the assessment tools used, the results of the assessment(s) (e.g., a functional hypothesis of challenging behavior, a preference hierarchy), and recommendations for the family and/or child. The ABA trainee also answers any questions regarding the functional and preference assessment processes and the rationale behind the provided recommendations and to set the stage for treatment planning if behavioral interventions are warranted. At the conclusion of all discipline evaluations and presentations, the trainee participates in a case conference with the team in which final diagnoses and recommendations are determined. The trainee may also assist in providing integrated results to the family following the full team evaluation offering an opportunity to share ABA strategies to be used in home, educational, or other therapeutic settings.

### Core Course

The Core Course training includes didactic and experimental content materials to increase knowledge in topics such as NDDs, life course and public health perspectives, family partnerships and family-centered practice, and selected populations and systems. The Core Course includes a total of 60 h of didactic training across the academic year. At the end of each semester, trainees write reflection essays on three Core Course sessions of their choice, sharing how they would apply the key messages from each session in their current or future practice, research, or advocacy experiences.

The ABA trainee participates in all Core Course experiences, which are opportunities to learn about other disciplines supporting people with NDD and various topics relative to leadership. As an additional leadership experience, the ABA trainee co-facilitates a Core Course session with their ABA Supplemental Faculty Member related to the history and foundations of ABA. A LEND graduate from the Advocacy discipline who identifies as Autistic has also collaborated on this presentation with the ABA trainee and Faculty Member. This has created an opportunity to discuss the field not only from the perspectives of ABA providers but also with a member of the autistic community. The presentation often leads to an open discussion among trainees and presenters regarding the positive and negative experiences of ABA, collaboration with other disciplines and self-advocates, and areas for growth within the field.

### Interdisciplinary Leadership Seminar (LEAD)

In the Leadership Series (60 h total), LEND faculty provide didactic and experiential content related to self-reflection of one’s own leadership journey and application to interdisciplinary teamwork and advanced leadership skills. Trainees complete the *StrengthsFinder self-assessment tool* early in the training year to model the expectation to build on the strengths of all team members, including those with lived experience of NDD. Faculty-led sessions discuss the importance of self-regulation and emotional intelligence in interactions with teams and patients/clients in healthcare settings. This course also provides content and experiences related to navigating crucial conversations, negotiation, conflict resolution, and administrative issues, and explores The Five Dysfunctions of a Team (Lencioni, 2002). Similarly, focused discussions on burnout and compassion fatigue are offered to support the long-term professional development and leadership capacity for these emerging professionals.

In the leadership series, the ABA trainee engages in individual and team-based experiential learning to build knowledge in all 12 MCH Leadership Competencies. Some examples of the activities in this series include Individualized Leadership Plan (ILP), Family Mentoring Project (FMP), Community Leadership Project (CLP), and Disciplinary Presentation. The ILP includes goals and action plans created by the ABA trainee which guide them in incorporating new knowledge and skills, based on the MCH Leadership Competencies, into daily practice (e.g., reading articles about culturally affirming ABA practices or taking the lead on coaching caregivers in clinical appointments). The trainee and their supervisor monitor the students’ progress together throughout the LEND training year using this living document.

To better understand the greater local community (i.e., families of a child with NDD and community-based organizations), the ABA trainee participates in the FMP and the CLP. From both experiences, the ABA trainee’s role is that of a learner, instead of the expert. The FMP allows the trainee to learn about a families’ experiences navigating the educational and healthcare systems through individual meetings and support group settings. The CLP involves volunteering with a community organization that provides direct services or supports to children with disabilities and their families. The trainee can also choose to observe Early Intervention providers as they implement evidence-based services in the home setting. These family-centered and community-based activities provide invaluable experiences and knowledge and encourage the ABA trainee to reflect on how to better serve the NDD community through family-centered care and appropriate resources.

Lastly, the Disciplinary Presentation provides an opportunity for the ABA trainee to educate LEND trainees from other disciplines on the training and role of a behavior analyst, while simultaneously providing clarity on any previous misconceptions of the profession. Ultimately, the experiences within the leadership series are designed to improve the ABA trainee’s leadership development through self-assessment, reflection, community-partnership, and mentorship, which in return, serves to improve the trainee’s current and future skills when working with the NDD community.

### Seminar in Evidence-Based Methods (SEBM)

The Seminar in Evidence-Based Methods (SEBM) allows the trainees to engage in various levels of research during their LEND training through interdisciplinary project teams. The ABA trainee collaborates with trainees and faculty from other disciplines on a research project. The ABA trainee’s role can vary depending on the SEBM project and the experiences of the trainee but may include writing an Institutional Research Board (IRB) research proposal, implementing different research methodology (e.g., interviews, focus groups, questionnaires), and analyzing and interpreting data. All trainee teams participate in the dissemination of their findings at the Ohio LEND Poster Symposium in partnership with the LEND program at The Ohio State University/Nisonger Center.

The following is an example of an SEBM research project and a description of a previous ABA trainee’s role within the project. The example SEBM research project utilized a family-centered perspective to better understand the lived experiences of people with a disability and their families in community settings. As part of their leadership goals, the ABA trainee led a focus group that included members from a hospital-based family advisory council to get community members’ feedback on what inclusion looks like in the community. Once the trainees had the community’s feedback, they used the information to guide the development of a survey related to inclusion in different settings. The ABA trainee, along with the other trainees, documented progress of the project and future directions via a poster presentation.

### Disciplinary Clinical Experiences

During the ABA trainee pilot year, the role did not include a clinical experience for two reasons. First, the COVID-19 pandemic resulted in substantially limited services during that time. Second, there was not enough time in the planning process to partner with a clinician who provided ABA-based services at the medical center. During the second year of the ABA training discipline, a supervised clinical experience was added for the ABA trainee. The trainee provided direct and/or consultative services in a Brief Intensive Behavior Therapy (BIBT) clinic with supervision from the ABA LEND Supplemental Faculty Member (BCBA-D) and a licensed psychologist who ran the clinic. This clinical experience enhanced the training program by enabling the ABA LEND trainee to directly apply the theoretical knowledge and leadership skills from the LEND curriculum. Additionally, it provided opportunities for the trainee to participate in meaningful discussion with trainees from various disciplines about their clinical training experiences.

The BIBT clinic provides direct services to children who demonstrate significantly challenging behaviors (e.g., children with aggressive and disruptive behaviors that are unsafe) and their families. Families attend clinic twice a week for 8 weeks, which aligns with the ABA trainee’s clinic schedule. The ABA LEND trainee engages in the clinical experience twice per week for a total of 8 h (4 h of direct and 4 h of indirect client work) and can follow multiple families throughout their time in clinic. To assist in developing clear and objective clinical goals while in the BIBT program, the ABA LEND trainee, Supplemental Faculty, and clinical supervisor use the trainee’s ILP, based on the MCH leadership competencies (e.g., cultural competency, teaching/coaching/mentoring, negotiation/conflict resolution) to guide the clinical training experience.

The ABA LEND training experience is comprehensive, where the ABA trainee has multiple opportunities to partake in various leadership roles throughout their training experience in the BIBT program that enhance their clinician skills. For example, throughout the 8 weeks when working with a client and their family, the trainee assesses the client’s behavior by developing, implementing, and coaching preference and functional behavior assessment protocols. Additionally, to enhance consultation skills, the ABA trainee learns to develop a family-centered approach by collaborating with caregivers to develop behavior treatment plans and increase generalization of behaviors outside of the clinic setting. There are also times where the ABA trainee can take a more indirect role and focus on improving their data collection and analysis skills before and during treatment, such as recording and graphing the frequency and duration of the client’s behavior. Another opportunity for the ABA trainee involves presenting at national conferences using the experiences and cases from their clinical experience.

### Training Experience Alignment with BCBA Competencies

The training experience of LEND ABA trainees aligns with training in each competency domain of the BCBA 6th Edition Test Content Outline List (BACB, 2023). Clinical supervision provides trainees opportunities to demonstrate skills across all domains. The specific domains in which the trainee receives training across the year depend on cases and clinical opportunities; however, to ensure proper supervision across domains, specific cases and activities may be selected to enhance the trainee’s supervised clinical experience. Interdisciplinary collaboration throughout the LEND program provides trainees with strong experiences in identifying information in records at the outset of the case, working with other disciplines to gather a broader range of relevant information (F.1.) and collaborating with others to enhance services (H.8.).

In addition to domains covered during the individual clinical training and supervision, the LEND program overall offers ABA trainees unique, interdisciplinary training to address specific task areas. For instance, as part of the LEND program the ABA trainee provides education on behaviorism and Applied Behavior Analysis through formal presentations to all trainees across disciplines and as a participant in interdisciplinary research, advocacy, and clinical activities. These activities include explaining the philosophical assumptions underlying behavior analysis (task list item A.2.), defining behavior from the perspective of radical behaviorism (A.3.), distinguishing experimental and applied behavior analysis and the professional practice of behavior analysis (A.4.), and explaining the dimensions of applied behavior analysis (A.5.). During the LEND presentations, the trainee also answers questions from colleagues from other disciplines about ABA and listens to their perspectives and experiences with ABA-based services and providers. This practice of active listening and responding to people with different perspectives with compassion and respect aligns with task list items E.1. (Identify and apply core principles underlying the ethics codes) and E.8. (Identify and apply interpersonal and other skills). Through the entire year of training, the ABA trainee can develop respectful and compassionate relationships with trainees and faculty from other disciplines, even when there initially may be conflict due to negative perspectives of ABA or unpleasant experiences with an ABA service or provider. A strong focus of the LEND program is understanding personal biases and how those biases impact professional work (E.11.). Additionally, trainees receive extensive training and feedback on cultural competency and humility throughout the LEND curriculum and activities (E.9.).

### Recommendations for Expansion of Behavior Analysis within LEND Training Programs

As many LEND programs may include behavior analysis trainees and faculty either within a behavior analysis specific track or within another discipline (e.g., psychology, special education), research on the current role of training about applied behavior analysis and inclusion of behavior analysts within LEND programs (e.g., how many of the didactics and activities include behavior analysts or have an ABA component) should be assessed. Additionally, for LEND programs that do not include behavior analysts consistently in their training programs, it will be important to understand the barriers for inclusion of behavior analysts and possible solutions to address these barriers which could include budgetary restrictions, lack of connection with behavior analysts in the area, or trainees from other disciplines, including parents and self-advocates who may have objections or concerns related to inclusion of professionals from ABA. LEND programs that currently include behavior analysts who participate under another training discipline such as psychology, speech and language pathology, occupational therapy, or special education, should assess if the creation of a behavior analysis specific training track would increase awareness of behavior analysis as its own discipline and allow for expansion of faculty and trainee to behavior analysts who do not have training in an additional discipline. Table 4 provides a list of possible guiding questions for inclusion of a behavior analysis specific track for LEND programs that do not currently have one.

**Table 4:** Guiding questions for addition of ABA discipline in LEND programs

Phase	Considerations
Initial planning	• Is there support from LEND leadership regarding the addition of a BA track in LEND? • Do we have a person(s) to be a clinical supervisor and/or LEND faculty? ○ Is there guidance to follow from MCHB regarding the criteria for who can serve as a supervisor or supplementary faculty? ○ Would there be any time or space conflicts? • What are the budgetary/funding needs for supervisor, supplementary faculty, and/or BA trainee? • Is there a need for current trainees to learn more about ABA and ABA providers? (solicitating feedback from current trainees) • Are there behavior analysists and/or behavior analysis students interested in being LEND trainees in an ABA specific training track (checking with local behavior analysts and behavior analysis higher education programs) • Is there a need in the community for the addition of a BA track? • How can we fully integrate the BA LEND track into LEND’s core components (e.g., BA trainee contributes FBA/preference assessment to the Interdisciplinary Training Team experience) • What might be objections or concerns with the LEND program or local community to adding a behavior analysis training discipline? • How would we know if adding a behavior analysis discipline benefitted our LEND training program? • How would we know if LEND training was beneficial to behavior analysis trainees?
Implementation	• How do we provide support to the BA LEND trainee and their supervisors? • How do we measure progress in the implementation of the new BA LEND track? • What type of data could be collected both quantitative and qualitative? • From whom are we collecting the data: the BA LEND trainee, trainees in other disciplines, supervisors/faculty, clients and/or other partners? • How often should we collect data? Formative vs. summative?
Evaluation	• Did we meet our goal in successfully creating and developing a BA LEND trainee track? • How do we know when the goal is met? • During the process of adding a BA LEND trainee track, what went well? What could be improved on? • How can our efforts be sustained in the following years?

Our program faced several of these barriers. Each year, we have had to evaluate and adjust the budget as needed to ensure a behavior analysis trainee and supplemental faculty in behavior analysis can be included in the program. To increase connections with local behavior analysts and trainees, we reached out to the ABA program at a local university and are continuing to develop that partnership. Our future work to increase connections with local behavior analysis will include reaching out to local ABA service providers to develop more potential partnerships within the community. Each year, we provide a LEND didactic training session on ABA in which all LEND trainees across disciplines are encouraged to share their knowledge and honest opinions and experiences with the field during the discussions. To date, we have collected de-identified informal survey feedback from LEND trainees about this didactic training session which has been overwhelmingly positive regarding increasing the knowledge of participants about ABA and addressing some previous misconceptions and understandings. Evaluating changes in perspectives of LEND Trainees from other disciplines with inclusion of an ABA specific track is an area for future research.

For behavior analysts seeking additional interdisciplinary training, determine if a LEND program is available in your region (https://mchb.hrsa.gov/programs-impact/focus-areas/mch-workforce-development/funded-project?train=285&inst=All&state=All&region=All&search =) and contact specific LEND programs to inquire about possibilities for training and/or collaboration. This could include partnering on research, advocacy, or community outreach as a first step in developing an increased relationship between behavior analysts within the community and local LEND programs.

## Summary

Recently, concerns regarding ABA services for people with NDD, especially autism, have been raised by clients, caregivers, and other professionals, including the ethics of using certain behavioral strategies (e.g., punishment, extinction) and the use of behavioral strategies to teach behaviors to have neurodivergent people appear neurotypical (e.g., Kupferstein, 2018; Sandoval-Norton & Shkedy, 2019). One important way we can better understand the experiences of people with NDD in ABA-based services is to develop relationships with programs that offer the opportunity for collaboration and critical reflections. By using a more person-centered, trauma-informed, and interdisciplinary approach, ABA providers can improve not only the public perception of ABA-based services, but, most importantly, the positive outcomes and experiences of clients they aim to serve (Rajaraman et al., 2021). Additionally, participation in interdisciplinary work and programs, such as LEND, increases the transparency of the work of ABA providers and can clarify misconceptions or misunderstandings that typically create barriers toward cross-disciplinary collaboration.

LEND programs have provided training to professionals in supporting people with NDD for over 60 years; however, many of these programs have not included professionals and trainees in the field of ABA (Health Resources & Services Administration, 2024). This has been a missed training opportunity for both LEND programs and ABA providers. At the beginning of each training year, there are often many trainees from other disciplines who know very little about ABA. Other trainees who have more knowledge or experiences with ABA-based services can share their experiences and perspectives on the field. Having an ABA trainee present during these discussions can be invaluable for clarifying misconceptions and for the trainee to practice active listening and empathy as others share their experiences.

LEND training includes many interdisciplinary training activities, including core course content across disciplines and interdisciplinary training clinical opportunities such as the ITT. Participation in a LEND program has been found to increase trainees’ skills and attitudes toward interdisciplinary work, as well as their leadership competencies (Smith et al., 2022; Weber et al., 2021). Partnering with existing interdisciplinary programs, such as LEND, can create opportunities for ABA trainees and professionals to increase their training and skills in interdisciplinary work and expand their knowledge about the field and role of behavior analysts in interdisciplinary care of people with NDD. To increase the presence of ABA within LEND programs, there first needs to be partnerships developed between ABA providers and LEND faculty. This can be accomplished through presentations to LEND trainees, collaborative research activities, or opportunities for LEND trainees and faculty to observe ABA-based services. Through these relationships, perhaps additional LEND ABA training opportunities can be developed.

## Data Availability

The current manuscript does not include any associated data.

## Declarations

### Author Note

The Leadership Education in Neurodevelopmental and related Disabilities Program (LEND) is funded through the Maternal and Child Health Bureau/Health Resources and Services Administration (HRSA) Grant No: T73MC00032.
